# Distinct mechanisms underlie electrical coupling resonance and its interaction with membrane potential resonance

**DOI:** 10.3389/fsysb.2023.1122433

**Published:** 2023-03-08

**Authors:** Xinping Li, Omar Itani, Dirk M. Bucher, Horacio G. Rotstein, Farzan Nadim

**Affiliations:** Department of Biological Sciences, New Jersey Institute of Technology, Newark, NJ, United States

**Keywords:** oscillation, central pattern generator, stomatogastric, gap junctions, resonance

## Abstract

Neurons in oscillatory networks often exhibit membrane potential resonance, a peak impedance at a non-zero input frequency. In electrically coupled oscillatory networks, the coupling coefficient (the ratio of post- and prejunctional voltage responses) could also show resonance. Such coupling resonance may emerge from the interaction between the coupling current and resonance properties of the coupled neurons, but this relationship has not been clearly described. Additionally, it is unknown if the gap-junction mediated electrical coupling conductance may have frequency dependence. We examined these questions by recording a pair of electrically coupled neurons in the oscillatory pyloric network of the crab *Cancer borealis*. We performed dual current- and voltage-clamp recordings and quantified the frequency preference of the coupled neurons, the coupling coefficient, the electrical conductance, and the postjunctional neuronal response. We found that all components exhibit frequency selectivity, but with distinct preferred frequencies. Mathematical and computational analysis showed that membrane potential resonance of the postjunctional neuron was sufficient to give rise to resonance properties of the coupling coefficient, but not the coupling conductance. A distinct coupling conductance resonance frequency therefore emerges either from other circuit components or from the gating properties of the gap junctions. Finally, to explore the functional effect of the resonance of the coupling conductance, we examined its role in synchronizing neuronal the activities of electrically coupled bursting model neurons. Together, our findings elucidate factors that produce electrical coupling resonance and the function of this resonance in oscillatory networks.

## 1 Introduction

In oscillatory circuits, neurons and synapses are subject to inputs that often span a range of frequencies. Whether they respond more favorably in one frequency range, and whether such frequency selectivity can be altered in different states, may impact the dynamics of the circuit output. Many neurons exhibit a frequency-dependent property known as membrane potential resonance, characterized as a maximal subthreshold impedance at a non-zero (resonance) frequency (Hutcheon and Yarom, 2000). When measured with oscillatory current injection, this corresponds to the voltage amplitude response being maximal to oscillatory current input at that frequency. Membrane potential resonance typically arises through interactions of passive properties of the neuron and the kinetics of voltage gated ionic currents (Hutcheon and Yarom, 2000). The resonance frequency of neurons has been shown to correlate with the network frequency in several systems (Wu et al., 2001; Bykhovskaia et al., 2004; Tohidi and Nadim, 2009; Moca et al., 2014). Membrane potential resonance is one form of preferred frequency response observed in neural circuits, but other circuit properties such as synaptic strengths and firing rate can also have a preferred frequency at which the output is maximized and such preferred frequencies are also often termed resonance (Izhikevich et al., 2003; Richardson et al., 2003; Drover et al., 2007; Ledoux and Brunel, 2011; Tseng et al., 2014; Rau et al., 2015; Stark et al., 2022).

In neural circuits coupled through gap junction-mediated electrical coupling, any input that causes membrane potential oscillations in one neuron could produce oscillations in its coupled partners (Landisman et al., 2002; Long et al., 2004). In electrically coupled networks where individual neurons exhibit membrane potential resonance, both the postjunctional neuron’s membrane potential and the coupling coefficient (the ratio of post- and prejunctional voltages) can also exhibit preferred frequency responses (Curti et al., 2012; Stagkourakis et al., 2018). However, it is not known if coupling resonance reflects the properties of the electrical coupling, those of the coupled neurons, or if it emerges from the interaction between the two. Electrical coupling is an important factor in generating neural oscillations (Posłuszny, 2014; Coulon and Landisman, 2017; Traub et al., 2018; Alcamí and Pereda, 2019) and, as we showed in a previous study, membrane potential resonance can directly influence the network oscillation frequency through electrical coupling (Chen et al., 2016). It is therefore important to understand how resonance properties of neurons can interact through electrical coupling.

We examined this question by recording pairs of electrically coupled neurons that show resonance in the oscillatory pyloric network of the crab, *Cancer borealis*. This circuit includes two bursting pyloric dilator (PD) neurons that are known to exhibit membrane potential resonance at a frequency close to the pyloric circuit oscillation frequency (Tohidi and Nadim, 2009; Fox et al., 2017). These two neurons are strongly electrically coupled to each other and, during normal activity, exhibit synchronous slow-wave oscillations that support their bursting activity (Marder and Eisen, 1984). We took advantage of the fact that we could examine the PD neurons’ membrane potential resonance and their coupling properties simultaneously to quantify the frequency dependent properties of the neurons, the coupling coefficient, and the coupling current (measured in voltage clamp). We found that all three components exhibit frequency selectivity, but with distinct preferred frequencies. Although resonance in the coupling coefficient has been previously reported, this is, to our knowledge, the first report of resonance in the coupling current.

We used mathematical analysis and computational modeling to explain the mechanism underlying resonance in the coupling coefficient, and what factors determine its resonance frequency. We then examined potential circuit mechanisms that may give rise to resonance in the coupling current and explored how such a resonance may influence network synchronization.

## 2 Materials and methods

### 2.1 Preparation and electrophysiology recordings

All experiments were performed on wild-caught adult male crabs (*Cancer borealis*) purchased from local seafood suppliers in Newark, NJ. Prior to experiments, animals were kept in artificial sea water tanks at 13 C. Before dissection, crabs were anesthetized by placing them on ice for 30 min. The STNS was dissected out following standard protocols (Blitz et al., 2004; Tohidi and Nadim, 2009), placed in a Petri dish coated with clear silicone elastomer (Sylgard 184; Dow Corning) and superfused with *C. borealis* saline, containing (in mM) 11 KCl, 440 NaCl, 13 CaCl_2_, 26 MgCl_2_, 11.2 Trizma base, and 5.1 maleic acid (pH = 7.4–7.5). A petroleum jelly well was built around the STG for constant superfusion of chilled (10–12°C) saline during the experiment.

PD neurons were identified by their characteristic intracellular waveforms and by matching their activities to the spikes on the corresponding motor nerves. Extracellular activities of motor nerves were recorded with a differential AC amplifier (Model 1700; A-M Systems), using stainless-steel pin wire electrodes placed inside and outside of small petroleum jelly wells built around the nerves. Intracellular recordings, current clamp and voltage clamp experiments were done with Axoclamp 900 A amplifiers (Molecular Devices). The STG was desheathed and the neuron cell bodies were impaled with sharp glass electrodes, prepared with a Flaming-Brown P-97 Puller (Sutter Instruments) and filled with 0.6 M K_2_SO_4_ + 20 mM KCl solution (15–30 MΩ electrode resistance). All electrophysiological data were digitized at 5–10 KHz with a Digidata 1440 A data acquisition board (Molecular Devices).

### 2.2 Measuring electrical coupling resonance and membrane potential resonance

We measured the membrane potential and electrical coupling resonance in pairs of PD neurons, in both current clamp experiments and voltage clamp experiments, with dual two-electrode recordings. In all experiments, we recorded the voltage in both the pre- and the postjunctional neurons (*V*_*pre*_ and *V*_*post*_) and the current injected into them (*I*_*pre*_ and *I*_*post*_). In current clamp experiments, a ZAP (Impedance Amplitude Profile) current was injected into the prejunctional neuron and produced oscillation in both *V*_*pre*_ and *V*_*post*_. The ZAP function was given by

$$\begin{array}{l} I_{ZAP}=I_{\max}\cos\left(2\pi f\left(t\right)\right)\\ f\left(t\right)=\frac{f_{\mathrm{lo}}}{L}\left(e^{Lt}-1\right)\\ L=\frac{1}{t_{\max}}\log\left(\frac{f_{\mathrm{hi}}}{f_{\mathrm{lo}}}\right) \end{array}$$

where *f*(*t*) swept a range of frequencies as a function of time, *t*, from *f*_lo_ = 0.1 Hz to *f*_hi_ = 4 Hz. *I*_max_ = 3 nA and produced a *V*_*pre*_ roughly ranging from −60 mV to −30 mV. *t*_max_ is the total duration of the ZAP waveform which, in most trials was at least 100 s. Additionally, to avoid transients, we always started the ZAP function with 2 pre-cycles of a sinusoidal current applied at the lowest frequency (*f*_lo_ = 0.1 Hz) that smoothly transitioned into the ZAP waveform. When measuring in voltage clamp, the same ZAP function was applied to the prejunctional voltage *V*_*pre*_ to force it to alternate between −60 and −30 mV, while the postjunctional neuron was held at a constant voltage of *V*_*post*_ = −60 mV. The prejunctional impedance (*Z*_*pre*_), the postjunctional impedance (*Z*_*post*_), the coupling coefficient (*CC*) and the coupling conductance (*G*_*c*_) were calculated as shown in Table 1.

**TABLE 1:** List of notations. All symbols in the table are functions of the input frequency *f*. The symbol $\hat{X}$ refers to the Fourier transform of *X*. In this manuscript we use the symbols below to denote the norm (|·|) of the complex values obtained by the Fourier transforms.

Function		Symbol	Definition	Postjunctional cell in
Impedance Amplitude of the Coupled Neuron (MΩ)	Prejunctional	*Z*_*pre*_	$\left|{\hat{V}}_{pre}/{\hat{I}}_{pre}\right|$	Either
	Postjunctional (current injected in *pre* neuron)	*Z*_*post*_	$\left|{\hat{V}}_{post}/{\hat{I}}_{pre}\right|$	Either
Impedance Amplitude of the *Isolated* Neuron (MΩ; neuron number *k =* 1 *or* 2; current injected in the same neuron)		*Z*_*k*_	$\left|{\hat{V}}_{k}/{\hat{I}}_{k}\right|$	Either
Coupling Coefficient (unitless)		*CC*	$\left|{\hat{V}}_{post}/{\hat{V}}_{pre}\right|$	Current clamp
Coupling Conductance (µS)		*G*_*c*_	$\left|{\hat{I}}_{post}/{\hat{V}}_{pre}\right|$	Voltage clamp

All factors measured as a function of frequency, in current or voltage clamp, were fit with a sixth-degree polynomial in MATLAB (The MathWorks Inc.) and the resonance frequency and amplitude were estimated as the peak amplitude of the fit curve and the frequency at which the maximum amplitude was achieved.

All experimental measurements involving electrical coupling were done in the presence of 100 nM tetrodotoxin citrate (TTX; Biotium) saline to block action potentials as well as the descending neuromodulatory inputs, and 5 µM picrotoxin (PTX; Sigma) to block chemical synapses within the STG, all of which are inhibitory.

### 2.3 Data and statistical analysis

All experimental data analysis was done using scripts written in MATLAB, and statistical comparisons were done in SigmaPlot 12 (SyStat Software Inc.). Critical significance level was set to *α* = 0.05. Unless otherwise indicated, all error bars in the figures represent standard error of the mean.

### 2.4 Model of coupled resonant neurons

We made biophysical models of coupled resonant neurons of Figures 4A, 5, using single compartment neurons having the Hodgkin-Huxley type currents given in Table 2. The model structure and parameters for the model neurons were implemented from the PD neuron resonance properties as previously described (Fox et al., 2017). Simulations were performed in NEURON 8.0 through the Python 3.8 interface. Analyses were conducted through custom Python scripts using scipy 1.5 and numpy 1.19 packages. All simulations for this study are available on https://github.com/fnadim/ECouplingResonance.

**TABLE 2:** Parameters of the coupled resonant neurons.

Cell	Current	Parameter	Value	Units
Model cells pre and post				
		*C*	2	nF
	Leak	*g*_*max*_	98	nS
			107.8 Cell 1 Figure 4Ci	
		*E*_*rev*_	−60	mV
	Ca	*g*_*max*_	100	nS
		*E*_*rev*_	120	mV
		*m*_*∞*_(v)	1/(1 + exp((v+52)/-7.2))	
		*p*	3	
		*τ*_*m*_(v)	40	ms
		*h*_*∞*_(v)	1/(1 + exp((v+60)/5))	
		*q*	1	
		*τ*_*h*_(v)	220 + 400* Ca_*h*_*∞*_(v)	ms
			1.8*(220 + 400* Ca_*h*_*∞*_(v)) Cell 1 Figure 4Ci	
	h	*g*_*max*_	60	nS
		*E*_*rev*_	−20	mV
		*m*_*∞*_(v)	1/(1 + exp((v+65)/4))	
		*p*	2	
		*τ*_*m*_(v)	1,500–1,400 * (1—h_*m*_*∞*_(v))	ms
			2,400–1,600 * (1—h_*m*_*∞*_(v)) Cell 1 Figure 4Ci	
Model cell 3		*C*	2	nF
	Leak	*g*_*max*_	30	nS
	Leak	*E*_*rev*_	−58	mV
	Ca	*g*_*max*_	12	nS
	Ca KS	*E*_*rev*_	120	mV
		*m*_*∞*_(v)	1/(1 + exp((v+55.56)/-3))	
		*p*	3	
		*τ*_*m*_(v)	8.95 + (58.37/(1 + exp((v+54.5)/3)))	ms
		*h*_*∞*_(v)	1/(1 + exp((v+60.12)/2))	
		*q*	1	
		*τ*_*h*_(v)	3155.4	ms
		*g*_*max*_	30	nS
	KS MI	*E*_*rev*_	−80	mV
		*m*_*∞*_(v)	1/(1 + exp((v+56)/-2))	
		*p*	2	
		*τ*_*m*_(v)	2000 + (−1,500/(1 + exp(-(v+55))))	ms
		*g*_*max*_	11	nS
	MI	*E*_*rev*_	−10	mV
		*m*_*∞*_(v)	1/(1 + exp((v+55)/-5))	
		*p*	1	
		*τ*_*m*_(v)	20	ms

### 2.5 Ball-and-stick model

The ball-and-stick model neurons were built using a point neuron, modeled as a sphere of diameter 100 μm, coupled to a single neurite of length 1,000 μm, divided into 101 compartments. Two forms of the neurite were used, as described in the Results. One was a standard cylinder of diameter 10 µm. The other was a tapered cylinder with diameter tapering (proximally-to-distally) linearly from 20 to 0.5 µm. The parameters used to build the model were R_m_ = 10000 Ωcm^2^, R_a_ = 100 Ωcm, Cm = 1 μF/cm^2^. For adding resonance, all compartments were modeled according to the parameters given in Table 2. Simulations were preformed in NEURON as described above.

### 2.6 Model of coupled bursting neurons

The model consisted of two neurons coupled with symmetric electrical coupling. Each neuron was built as a two-compartment biophysical model, consisting of a soma/neurite (*SN*) and an axon (*A*) compartment. The soma/neurite compartment included a leak and a low-threshold (T-type) inactivating calcium current, which effectively made it a calcium spike oscillator (Torben-Nielsen et al., 2012). The axon compartment included Hodgkin-Huxley type leak, fast sodium and delayed rectifier potassium currents, which allowed it to spike but only when the input from the soma/neurite compartment produced a calcium spike. The combination produced a bursting neuron. The neuron obeyed the following standard Hodgkin-Huxley type current balance equations:

$$\begin{array}{l} C_{SN}\frac{dV_{SN}}{dt}=I_{L-SN}+I_{Ca}+I_{axial}+I_{c}\\ C_{A}\frac{dV_{SN}}{dt}=I_{L-A}+I_{Na}+I_{K}-I_{axial} \end{array}$$

where *C*_*x*_ and $I_{L-x}=g_{L-x}\left(V-E_{L-x}\right)$ denote the membrane capacitance and leak current of the compartments (*x = SN* or *A*), $I_{axial}=g_{axial}\left(V_{SN}-V_{A}\right)$ is the axial current between the two compartments. The electrical coupling current is $I_{c}=G_{c}\left(V_{SN}-V_{SN2}\right)$ , where *V*_*SN2*_ is the voltage of the other neuron’s *SN* compartment and *G*_*c*_ is the coupling conductance, which may be frequency-dependent (see below). The ionic currents are given as

$$I_{ion}={\bar{g}}_{ion}m_{ion}^{p}h_{ion}^{q}\left(V-E_{ion}\right)$$

where *ion* = *Ca*, *Na* or *K*, ${\bar{g}}_{ion}$ is the maximal conductance, and *m*_*ion*_ and *h*_*ion*_ denote the activation and inactivation gating variables governed by

$$\frac{dx}{dt}=\frac{1}{\tau_{x}}\left[x_{\infty }\left(V\right)-x\right]$$

(*x* = *m*_*ion*_ or *h*_*ion*_). The activation and inactivation powers, *p* and *q*, are non-zero integers. The model equations and parameters are provided in Table 3. The parameters of the two neurons were chosen so that, in isolation, their bursting frequencies differed by about 10%.

**TABLE 3:** Parameters of the coupled bursting neurons. a_1_, b_1_ and c_1_ are scaling parameters. For cell 1, a_1_ = 0, b_1_ = 0, c_1_ = 1; for cell 2, a_1_ = 0.412241, b_1_ = −0.0282679, c_1_ = 1.125. All capacitances in pF, conductances in nS, time constants in ms.

Compartment	Current	Parameter	Value
		*g*_*axial*_	130 nS
Soma/Neurite		*C*	2 nF
	Leak	*g*_*max*_	95 nS
		*E*_*rev*_	−63 mV
	Ca	*g*_*max*_	70 nS
		*E*_*rev*_	60 mV
		*m*_*∞*_(v)	1/(1 + exp(−0.4*(v+59.7-b_1_)))
		*p*	3
		*τ*_*m*_(v)	15 + 25*(1-Ca_*m*_*∞*_(v))
		*h*_*∞*_(v)	1/(1 + exp(0.8*(v+60-b_1_)))
		*q*	1
		*τ*_*h*_(v)	150 + 190/(1 + exp(0.1*(v+60-b_1_)))
Axon		*C*	250 pF
	Leak	*g*_*max*_	5 nS
		*E*_*rev*_	−65 mV
	Na	*g*_*max*_	3000 nS
		*E*_*rev*_	50 mV
		*m*_*∞*_(v)	1/(1 + exp(−0.085*(v+22)))
		*p*	3
		*τ*_*m*_(v)	0 (instantaneous)
		*h*_*∞*_(v)	1/(1 + exp(0.12*(v+30)))
		*Q*	1
		*τ*_*h*_(v)	2 ms
	K	*g*_*max*_	500 nS
		*E*_*rev*_	−80
		*m*_*∞*_(v)	1/(1 + exp(−0.15*(v+20)))
		*p*	4
		*τ*_*m*_(v)	2 + 14*(1-K_*m*_*∞*_(v))

The *G*_*c*_ frequency dependence was modeled to show resonance at *f* = 0.75 Hz according to the following equation:

$$G_{c}=2.625{\bar{G}}_{c}\left[e^{-0.1f}-e^{-5f}\right]$$

where 2.625 is a scaling factor so that $G_{c}={\bar{G}}_{c}$ at the resonance frequency.

Simulations were done in C, using a 4th order Runge-Kutta numerical integrator. The two cells always started with identical initial conditions and each run was 25 s. A 15 s window, ending 1 s before the simulation end (to remove filtering artifacts), was used for measurements of synchrony. The two voltage waveforms were sampled at 1 KHz The Slow waveform was obtained by low-pass filtering the waveforms with a moving average window of length 81 ms. The Fast waveform was obtained as the difference between the Full waveform and the Slow waveform. The level of synchrony was measured as, *R* ^2^, the square of the correlation coefficient between the (Full, Slow or Fast) waveforms of the two cells in this time window. All analysis was done in MATLAB.

## 3 Results

### 3.1 The coupling coefficient between the PD neurons exhibits resonance at a distinct frequency from their membrane potential resonance

**FIGURE 1:**
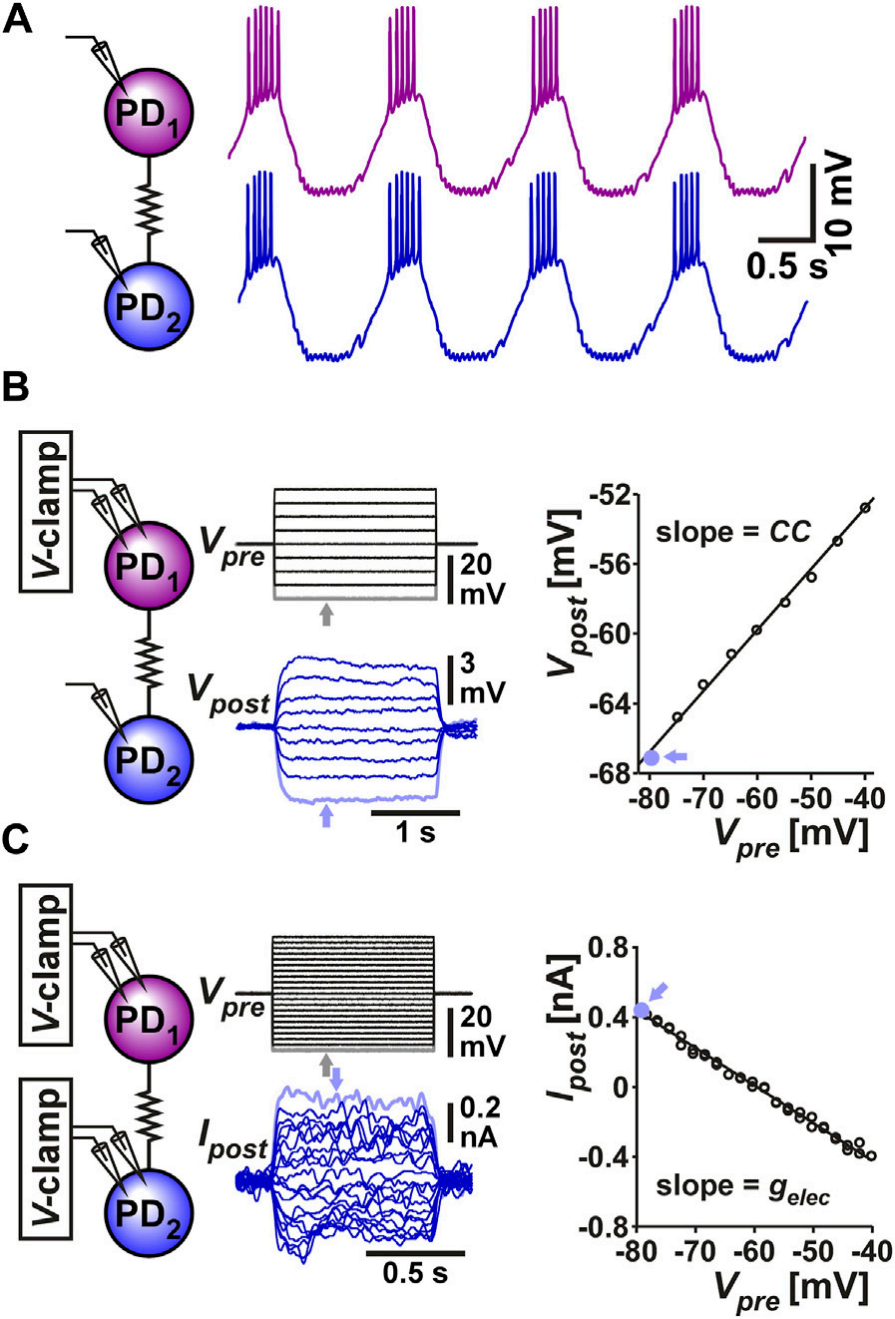
The two PD neurons produce synchronized slow wave bursting due to their strong electrical coupling. **(A)** Somatic recording of the two PD neurons shows that they produce bursting oscillations that are synchronized in their slow-wave activity. **(B)** Measurement of coupling coefficient between the two PD neurons. The prejunctional PD_1_ neuron is voltage clamped with steps ranging from −80 to −40 mV from a holding potential of −60 mV. The postjunctional PD_2_ neuron membrane potential is recorded in current clamp. The coupling coefficient *CC* is measured as the slope of the linear fit to the values of *V*_*post*_ plotted vs. *V*_*pre*_. Each data point is the mean value of voltage during the step, as seen in the grey point, corresponding to the lowest steps (arrows). **(C)** Measurement of coupling conductance between the two PD neurons. The prejunctional PD_1_ neuron is voltage clamped as in panel B, while the postjunctional PD_2_ neuron is voltage clamped at a steady holding potential of −60 mV (not shown). The coupling conductance *G*_*c*_ is measured as the slope of the linear fit to the values of *I*_*post*_ plotted vs. *V*_*pre*_. Each data point is the mean value the step, as seen in the grey point, corresponding to the lowest *V*_*pre*_ and highest *I*_*post*_ steps (arrows).

The two PD neurons are very similar in their ionic current expression and anatomical structure and therefore considered to be functionally equivalent, if not identical (Marder and Eisen, 1984; Bucher et al., 2005; Schulz et al., 2006). During normal pyloric activity, these two neurons exhibit synchronous slow-wave oscillations that support their bursting activity (Figure 1A). This synchronous activity arises primarily from their electrical coupling to one another and to the pyloric pacemaker, the anterior burster (AB) neuron (Marder and Eisen, 1984). The electrical coupling strength between the two PD neurons can be determined in the classical way as the coupling coefficient (*CC*), measured as the ratio of the voltage change of the postjunctional neuron to that of the prejunctional neuron (Figure 1B):

$$CC=\frac{\Delta V_{post}}{\Delta V_{pre}}.$$

A more direct measure of the strength of coupling, which does not depend on the input resistance of the postjunctional neuron can be obtained by voltage clamping both neurons, stepping the voltages of the (arbitrarily-designated) prejunctional neuron and measuring the current flow to the postjunctional cell. The coupling conductance (*G*_*c*_) can be measured as (Figure 1C):

$$G_{c}=\frac{\Delta I_{post}}{\Delta V_{pre}}.$$

**FIGURE 2:**
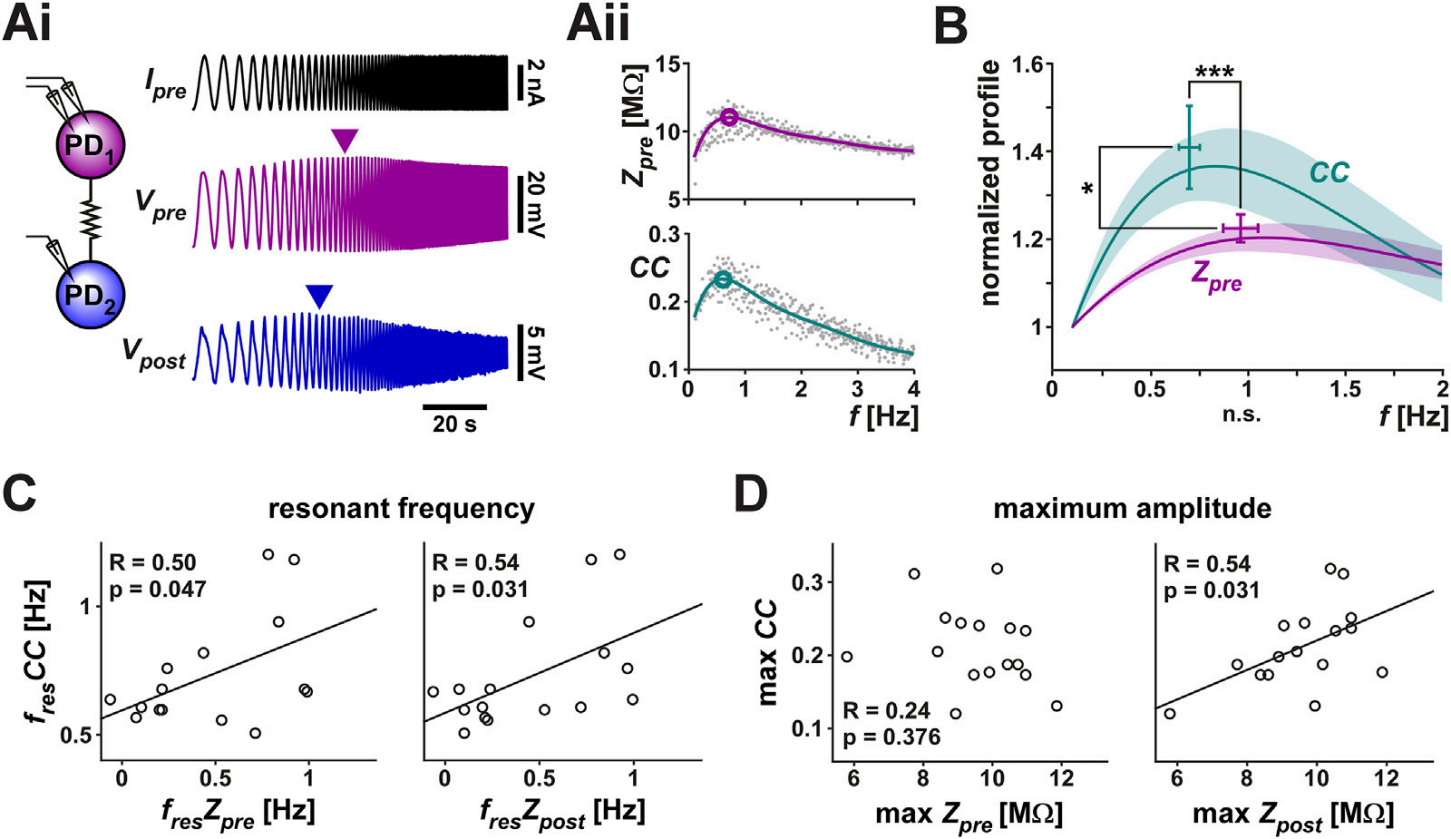
The coupling coefficient (*CC*) between the two PD neurons shows resonance. A ZAP current, sweeping a frequency range of 0.1–4 Hz, was applied to one PD neuron to simultaneously measure the voltage changes in both PD neurons. **(Ai)** Both neurons showed a peak amplitude response at an intermediate frequency (marked by arrowheads). Schematic shows the two coupled neurons monitored in current clamp. **(Aii)** The prejunctional impedance (*Z*_*pre*_) and *CC* of the data shown in Ai. A 6th order polynomial fit (smooth curves) to the raw data was used to measure the peak amplitude and resonance frequency (circled). **(B)** *Z*_*pre*_ and *CC* have distinct resonances. Averaged frequency profiles of *CC* and *Z*_*pre*_ are shown, both normalized to their amplitude at 0.1 Hz. *CC* had a smaller resonance frequency than *Z*_*pre*_ (*p* < 0.001) and higher resonance power (*p* = 0.037). *N* = 19, paired Student’s t-test. **(C, D)** The resonance frequency of *CC* was correlated with the resonance frequency of both *Z*_*pre*_ and *Z*_*post*_ **(C)**, while its maximum amplitude was only correlated with that of *Z*_*post*_ **(D)**.

The PD neurons are bursting oscillators and, additionally, these neurons show membrane potential resonance at a frequency correlated with their burst frequency (Tohidi and Nadim, 2009; Tseng and Nadim, 2010; Fox et al., 2017). We were interested in knowing whether the coupling strength between the two PD neurons (the PD-PD coupling) depends on, or is influenced by, their oscillation frequency and, if so, if the coupling also shows resonance. In the context of this manuscript, resonance is defined as a neuronal property that produces a maximum response to oscillatory input at a non-zero frequency. To compare any frequency dependence of the electrical coupling and that of the individual neurons, it was necessary to measure these two factors simultaneously. To do so, we arbitrarily designated the two PD neuron as pre- and postjunctional, injected a sweeping-frequency sinusoidal (ZAP) current into the prejunctional PD neuron and measured the voltage responses in both pre-and postjunctional PDs (Figure 2A). We then switched the pre and post designations and repeated the protocol. In the trials shown here, the ZAP function frequency is swept from 0.1 to 4 Hz, a range that covers the natural burst frequency of PD neurons which is typically between 0.5 and 2.5 Hz. In several trials we also changed the direction of the frequency sweep to go from high to low frequency. There was no difference in our measurements when the direction of the sweeping frequency of the ZAP current was reversed.

In 19 out of 28 measurements, both the prejunctional membrane impedance (*Z*_*pre*_; Table 1) and the coupling coefficient (*CC*) showed clear resonance (Figure 2B). Note that the peak values shown in the figure do not exactly match the peak of the mean profile (solid line) since the peak of the average of multiple non-linear curves is determined by the overall shapes of the individual curves, not just by their peaks. In response to the ZAP current, however, *Z*_*pre*_ and *CC* showed distinct frequency profiles (Figure 2B): *CC* had a lower resonance frequency (0.70 ± 0.20 Hz) than *Z*_*pre*_ (0.97 ± 0.36 Hz) and the normalized peak amplitude of *CC* was larger than that of *Z*_*pre*_. Additionally, the resonance frequency of *CC* was correlated with the resonance frequency of both the prejunctional and postjunctional impedance (*Z*_*pre*_ and *Z*_*post*_, Figure 2C), while its maximum amplitude was only correlated with that of *Z*_*post*_ (Figure 2D).

### 3.2 Electrical coupling conductance shows a preferred frequency (resonance)

Membrane potential resonance can be measured using both current clamp and voltage clamp methods, each providing its own advantage. Current clamp measurements allow the membrane potential to change freely and therefore, voltage-dependent ionic currents can also influence the membrane potential. This method allows one to observe neuronal responses in a manner closer to their natural biological activity and, in general, current clamp measurements provide a more realistic value of the impedance amplitude (Rotstein and Nadim, 2019). However, because the electrical coupling coefficient is influenced by the input resistance of the postjunctional neuron, it is not a direct measure of the strength of electrical coupling (Bennett, 1966; Mann-Metzer and Yarom, 1999). A direct estimate of the electrical coupling conductance, *G*_*c*_, requires measuring the current flowing between the two coupled neurons (Table 1) and, to obtain an accurate measurement of the ionic current, the membrane potentials must be constrained using the voltage clamp method, as we showed in Figure 1C.

**FIGURE 3:**
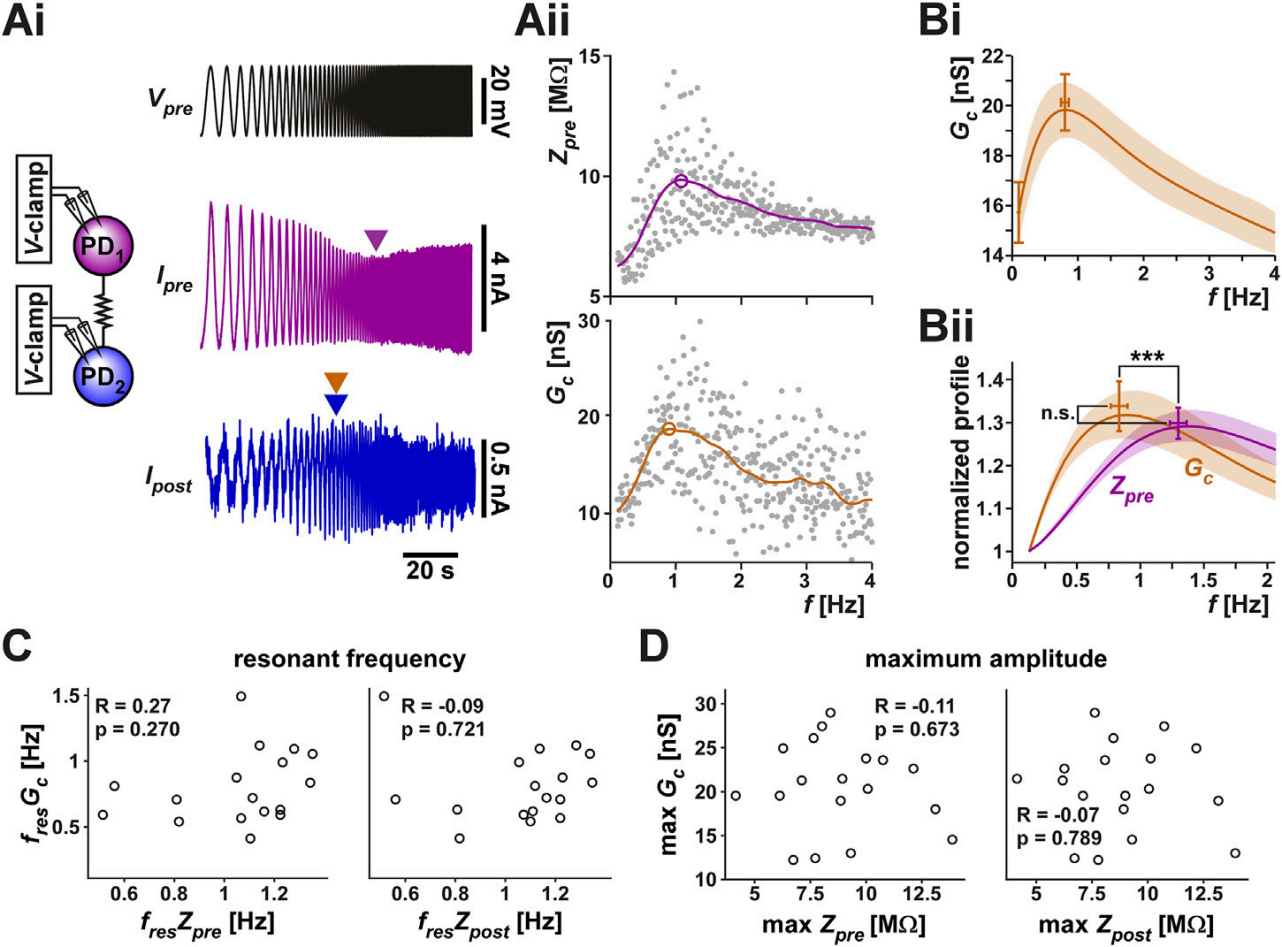
The coupling conductance shows a frequency-dependent resonance which is distinct from the resonance of the coupled PD neurons. **(Ai)** The two PD neurons were voltage clamped, the prejunctional neuron with a ZAP waveform, sweeping a frequency range of 0.1–4 Hz and a voltage range of −60 to −30 mV, while the postjunctional neuron was held at constant holding potential of −60 mV (not shown), and the current flow in both neurons was measured. *I*_*pre*_ showed a minimum value at an intermediate frequency, reflecting the intrinsic resonance of the prejunctional neuron (magenta arrowhead), while *I*_*post*_ showed a peak at a distinct frequency (blue/bronze arrowheads). Schematic represents the two coupled neurons in voltage clamp. **(Aii)** The prejunctional impedance (*Z*_*pre*_) and *G*_*c*_ measured from the data shown in **Ai**. A 6th order polynomial fit (smooth curves) to the raw data was used to measure the peak amplitude and resonance frequency (circled). The peak of *G*_*c*_ corresponds to the bronze color arrowhead in **(Ai)**. **(Bi)** The frequency profile of *G*_*c*_ across experiments shows a peak below 1 Hz. **(Bii)** *Z*_*pre*_ and *G*_*c*_ have distinct resonances. Averaged frequency profiles of *G*_*c*_ and *Z*_*pre*_ are shown, both normalized to their amplitude at 0.1 Hz. *G*_*c*_ had a smaller resonance frequency than *Z*_*pre*_ (*p* < 0.001) but comparable resonance power *Z*_*PD*_ (*p* = 0.525). *N* = 20, paired Student’s t-test. **(C, D)** Neither the resonance frequency **(C)**, nor the resonance amplitude **(D)** of *G*_*c*_ was correlated with that of *Z*_*pre*_ or *Z*_*post*_.

To directly measure whether the coupling conductance *G*_*c*_ is influenced by frequency, we voltage clamped both PD neurons at a holding potential of −60 mV. We then applied a ZAP function voltage waveform (ranging from −60 to −30 mV) to the prejunctional neuron, while holding the postjunctional neuron at a steady voltage of −60 mV (Figure 3Ai). This allowed us to simultaneously measure the currents flowing in the pre- and postjunctional neurons (*I*_*pre*_ and *I*_*post*_) in response to the change in the frequency of *V*_*pre*_. As seen in the example in the figure, *I*_*pre*_ showed a clear minimum in response to the voltage ZAP, indicating a minimum in the neuronal admittance (the reciprocal of impedance) value. This simply reflects the membrane potential resonance in the prejunctional PD neuron as measured in voltage clamp (Tseng and Nadim, 2010) (Figure 3Aii, top panel). Interestingly, in response to the prejunctional ZAP function, the postjunctional current, *I*_*post*_, did not remain constant in amplitude but had a clear maximum amplitude at a non-zero frequency. Therefore, the PD-PD coupling conductance, *G*_*c*_, also showed a peak at this frequency (Figure 3Aii, bottom panel).

Unlike the measurements with the step protocol, in which the directionality of the electrical coupling had little influence, we found that the two directions of the coupling often produced slightly different results. Therefore, in this part of the study, we treated the PD1 to PD2 and the PD2 to PD1 in each preparation independently. In 20 of the 28 measured cases, *G*_*c*_ showed resonance. Figure 3Bi shows the averaged resonance profile of these 20 electrical connections.

Because *Z*_*pre*_ and *G*_*c*_ have different units, their amplitudes cannot be directly compared. Yet it is useful to examine how much larger each of these factors is at its peak compared to its baseline. In fact, membrane potential resonance power is often measured as a ratio of the peak impedance *Z*_max_ to the impedance at zero frequency (i.e., the input resistance). We used the values of *Z*_*pre*_ and *G*_*c*_ at the lowest frequency (0.1 Hz) as a proxy for the zero-frequency values and normalized these curves to this value for each experiment (Figure 3Bii). A paired comparison between *G*_*c*_ and the impedance profile (*Z*_*pre*_; see Table 1) of the prejunctional neuron showed no difference in their relative amplitudes. However, *G*_*c*_ had a significantly lower resonance frequency (0.80 ± 0.26 Hz) than *Z*_*pre*_ (1.27 ± 0.23 Hz). Also, note that the resonance frequencies for *Z*_*pre*_ were different between current clamp and voltage clamp experiments, because, as described above, *Z*_*pre*_ measured in current clamp is influenced by non-linear actions of voltage-gated ionic currents. Finally, unlike with the coupling coefficient *CC*, we did not observe any correlation between *Z*_*pre*_ or *Z*_*post*_ and *G*_*c*_ either in frequency (Figure 3C) or in amplitude (Figure 3D). This is consistent with the hypothesis that *G*_*c*_ reflects the properties of the electrical coupling and not those of the coupled neurons.

### 3.3 Modeling elucidates how resonance of the coupling coefficient *CC* arises

**FIGURE 4:**
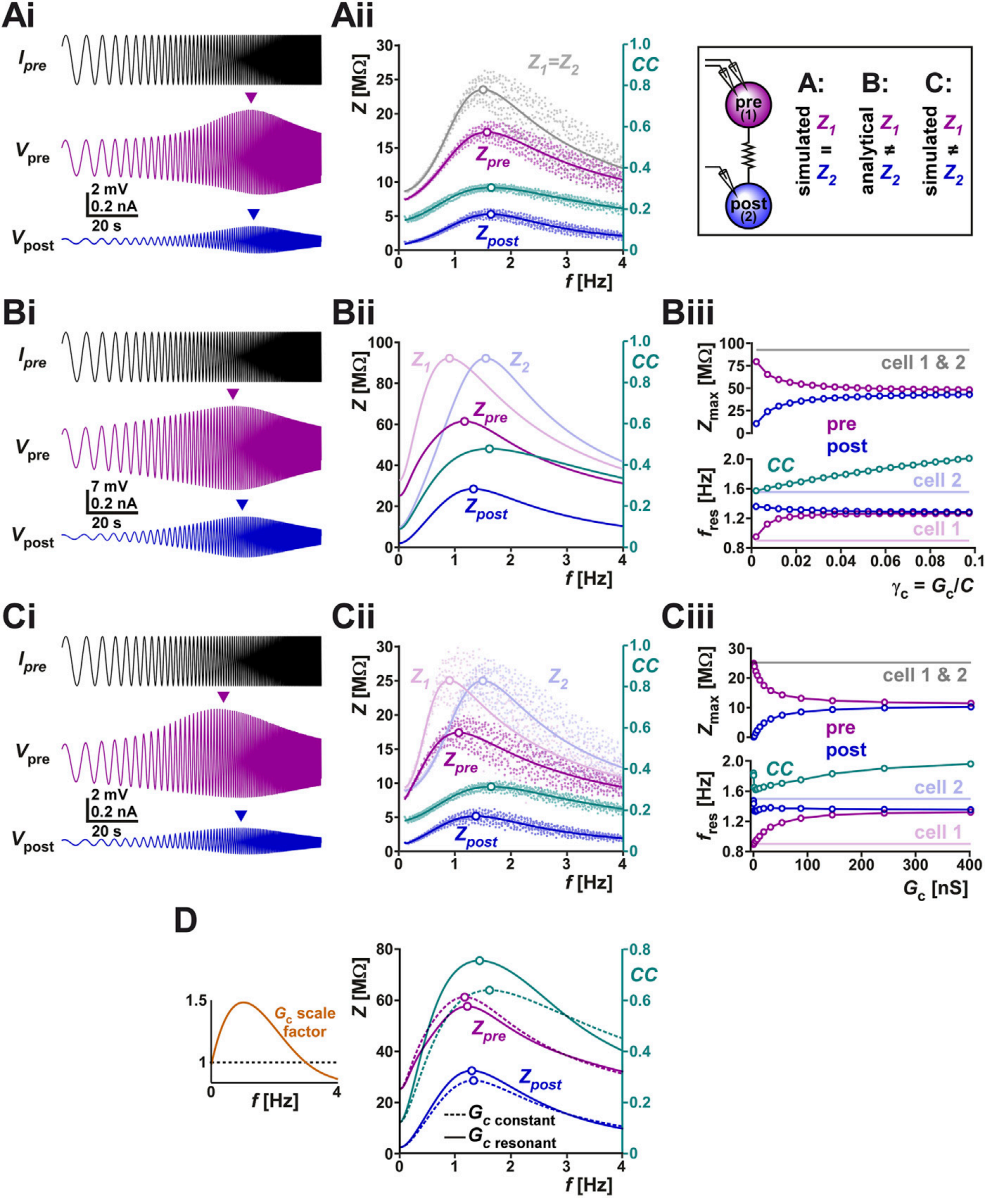
**(A)** The right panel schematically shows the protocols of this figure, in which we examine the current-clamp responses of two coupled identical biophysical model neurons **(A)**, two coupled linear resonators with distinct resonance frequencies measured analytically **(B)** and two coupled biophysical model neurons with distinct resonance frequencies **(C)**. **(Ai)** Two identical biophysical model neurons (parameters in Table 2) were coupled (*G*_*c*_ = 20 nS) and a ZAP current sweeping frequencies of 0.1–4 Hz was injected in both neurons to measure the changes in their membrane potentials. Arrows in Ai show the peak (resonance) values in membrane potential amplitudes. (**Aii)** Membrane impedance amplitudes of the pre- and postjunctional model neurons (*Z*_*pre*_ and *Z*_*post*_, respectively) of panel **(Ai)** shown in raw form (dots) and with a non-linear curve fit (solid curves). The impedance profile of the isolated identical pre- and post-junctional cells (1 and 2) are also shown in gray. The coupling coefficient (*CC*) shows a resonance frequency at a value close to those of *Z*_*pre*_ and *Z*_*post*_. The peak (resonance) frequencies are shown as open circles. **(Bi)** Simulation of a ZAP current injected in one of two coupled linear resonators, shown for comparison with the biophysical simulations. **(Bii)** Analytical calculations (see Supplementary Appendix SA1) show that coupling two linear resonators with distinct resonance frequencies (shown in Bi) brings the resonance peaks (open circles) closer to each other. *Z*_*pre*_ and *Z*_*post*_ show the impedance amplitude profiles of the coupled neurons whose isolated impedance profiles are shown in *Z*_1_ and *Z*_2_. In contrast, resonance frequency of *CC* does not fall between those of *Z*_1_ and *Z*_2_. **(Biii)** Bottom panel shows the resonance frequencies (*f* values of open circles in Bi) as a function of increasing coupling conductance *γ*_*c*_ (=*G*_*c*_ */C*; *C* is the membrane capacitance). Top panel shows the resonance amplitudes (*Z* values of open circles in Bii). **(C)** Panels **(Ci–Ciii)** show simulations of two coupled biophysical neurons [as in **(A)**], confirming the findings of the analytical model (panel B). Panel descriptions are the same as in **(B)**. Cell 1 was made to have a different resonance frequency by adjusting the parameters as indicated in Table 2. Cell 2 is identical to that of panel **(A)**. **(D)** The linear model is used to compare the effect of *G*_*c*_ resonance on the coupling coefficient CC. The left panel shows the two cases compared. In one (scale factor of 1), *G*_*c*_ is kept constant whereas in the other *G*_*c*_ is scaled by an inverted U function, mimicking the resonance measured experimentally in Figure 3 B. The right panel compares *Z*_*pre*_, *Z*_*post*_ and *CC* for the two cases, using the linear models of panel **(B)**. Note the amplification of *CC* and the shift in its resonance frequency when *G*_*c*_ shows resonance.

Frequency dependence of electrical coupling may emerge from the properties of the coupled neurons, may be a property of the junctional coupling itself, or arise from the interaction of the two. To demonstrate how resonance of the coupling coefficient *CC* could arise from the membrane potential resonance properties of the coupled neurons, we coupled two biophysical models that capture the resonance properties of the isolated PD neuron (Fox et al., 2017) with a constant electrical coupling coefficient (parameters for the model given in Table 2). We injected a ZAP current into one neuron and measured the voltage responses of both neurons (Figure 4Ai). Current injection to PD model neuron 1 resulted in membrane potential resonance, mainly due to the intrinsic properties of this neuron, and current flow through the electrical coupling to PD model neuron 2 produces membrane potential resonance in the second neuron. In this simulation, the two PD model neurons were identical and therefore, when isolated, had identical impedance profiles (*Z*_1_ = *Z*_2_ in Figure 4Aii). Coupling only slightly changed the impedance profile of either neuron compared to its profile when isolated (*Z*_*pre*_ compared to *Z*_1_; *Z*_*post*_ compared to *Z*_2;_ see Table 1 for notations). In this simulation, the *CC* vs. frequency curve also showed resonance, with a resonance peak frequency at a value close to, but different from, that of the coupled neurons. This gave rise to the question what factors determine the resonance frequency of *CC*.

To address this question, we switched to linear resonator neurons in which the impedance profile can be mathematically calculated (Richardson et al., 2003; Rotstein and Nadim, 2014). The full analysis is provided in Supplementary Appendix SA1. In the linear system of two coupled resonator neurons, the value of the coupled impedance profiles, as a function of the respective uncoupled profiles is given by

$$\begin{array}{l} Z_{pre}=\frac{{Z_{2}}^{-1}+G_{c}}{\left({Z_{1}}^{-1}+G_{c}\right)\left({Z_{2}}^{-1}+G_{c}\right)-G_{c}^{2}}\\ Z_{post}=\frac{G_{c}}{\left({Z_{1}}^{-1}+G_{c}\right)\left({Z_{2}}^{-1}+G_{c}\right)-G_{c}^{2}}. \end{array}$$

(Supplementary Equation SA1.8 of the Appendix with notations of Table 1) and the value of *CC* reduces to the ratio of the amplitudes of the two impedance profiles.

$$CC=\frac{Z_{post}}{Z_{pre}}=\frac{G_{c}}{\left\|{Z_{2}}^{-1}+G_{c}\right\|}.$$ (1.1)

Here, **Z**_2_ is the complex impedance profile of the postjunctional neuron when isolated (*Z*_2_ is the amplitude of the complex **Z**_2_, i.e., *Z*_2_ = ||**Z**_2_||). Note that for linear resonator neurons, *CC* only depends on the impedance of the (isolated) postjunctional neuron and not on that of the prejunctional neuron. Although this result does not generally hold for non-linear (e.g., biological) resonators, it still provides a very good approximation in most cases, in addition to a clearer conceptual understanding of the phenomenon.

The coupled linear resonators provide insight into how electrical coupling influences the resonance properties of the neurons as well as that of *CC*. For instance, the simulation of two electrically coupled linear resonators (as described in Supplementary Appendix SA1) and injected with a ZAP current shows distinct peak amplitudes in the pre- and post-junctional neuron’s membrane potentials (Figure 4Bi). In fact, coupling two linear resonators with the same maximal amplitude, but distinct resonance frequencies, shifted the resonance frequencies of both neurons toward values in between those of the isolated neurons (Figure 4Bii; compare peak frequencies of *Z*_*pre*_ and *Z*_*post*_ with *Z*_1_ and *Z*_2_). The resonance frequency *Z*_*post*_ fell between *Z*_*pre*_ and *Z*_2_. The postjunctional impedance profile (*Z*_*post*_: which is *V*_*post*_/*I*_*pre*_ in Figure 4Ai; see Table 1 for definition) always had a lower amplitude than the prejunctional profile (compare *Z*_*pre*_ and *Z*_*post*_ in Figure 4Bii). In Figure 4Bii, we also show the frequency-dependent profile of *CC* for comparison (note the different scales). Here, the resonance frequency of *CC* was close to that of *Z*_2_.

Interestingly, however, the resonance frequency of *CC* was not constrained to fall between the resonance frequencies of *Z*_1_ and *Z*_2_. This can be readily observed using the linear resonator models when current was injected in the cell with lower resonance frequency (cell 1 in Figure 4Bii) and the electrical coupling conductance was increased. When the electrical coupling conductance was small, the resonance frequency of *CC* was close to that of *Z*_2_, but when *G*_*c*_ was increased, this frequency also increased monotonically (Figure 4Biii bottom panel). Not surprisingly, increasing the strength of coupling also caused the resonance frequencies (Figure 4Biii bottom panel) and maximum pre- and postjunctional impedance values (Figure 4Biii top panel) to converge to the same value. These predictions of the analytical linear models can be confirmed by simulating a biophysical model of two coupled resonator neurons. Figure 4Ci shows such a simulation, which is based on the same neurons as in panel Ai, except that the prejunctional neuron’s parameters have been changed to allow for a lower resonance frequency while keeping the same resonance amplitude. The findings for *Z*_*pre*_, *Z*_*post*_ and *CC* (Figure 4Cii) are qualitatively similar to those of the linear model, as is the dependence of the resonance frequencies and peak resonance values of these attributes as a function of *G*_*c*_ (Figure 4Ciii). The only small difference between the biophysical and linear models arises at very small *G*_*c*_ values, where, in the biophysical model (Figure 4Ciii), *Z*_*post*_ and *CC* show a non-monotonic dependence on *G*_*c*_.

One can also use the coupled linear resonators to predict how a frequency-dependent *G*_*c*_ influences the measured coupling coefficient. To make this comparison, we scaled *G*_*c*_ as a function of frequency in a manner similar to what we had measured in the biological system (Figure 3Bii and left panel of Figure 4D). A comparison of the resulting *CC* and the *CC* obtained with a constant *G*_*c*_ value across frequencies showed that the resonance frequency of *G*_*c*_ can clearly amplify the amplitude of *CC*, by bringing the *Z*_*pre*_ and *Z*_*post*_ curves closer to each other in this range (Figure 4D right panel).

### 3.4 Can coupling conductance resonance result from network connectivity?

**FIGURE 5:**
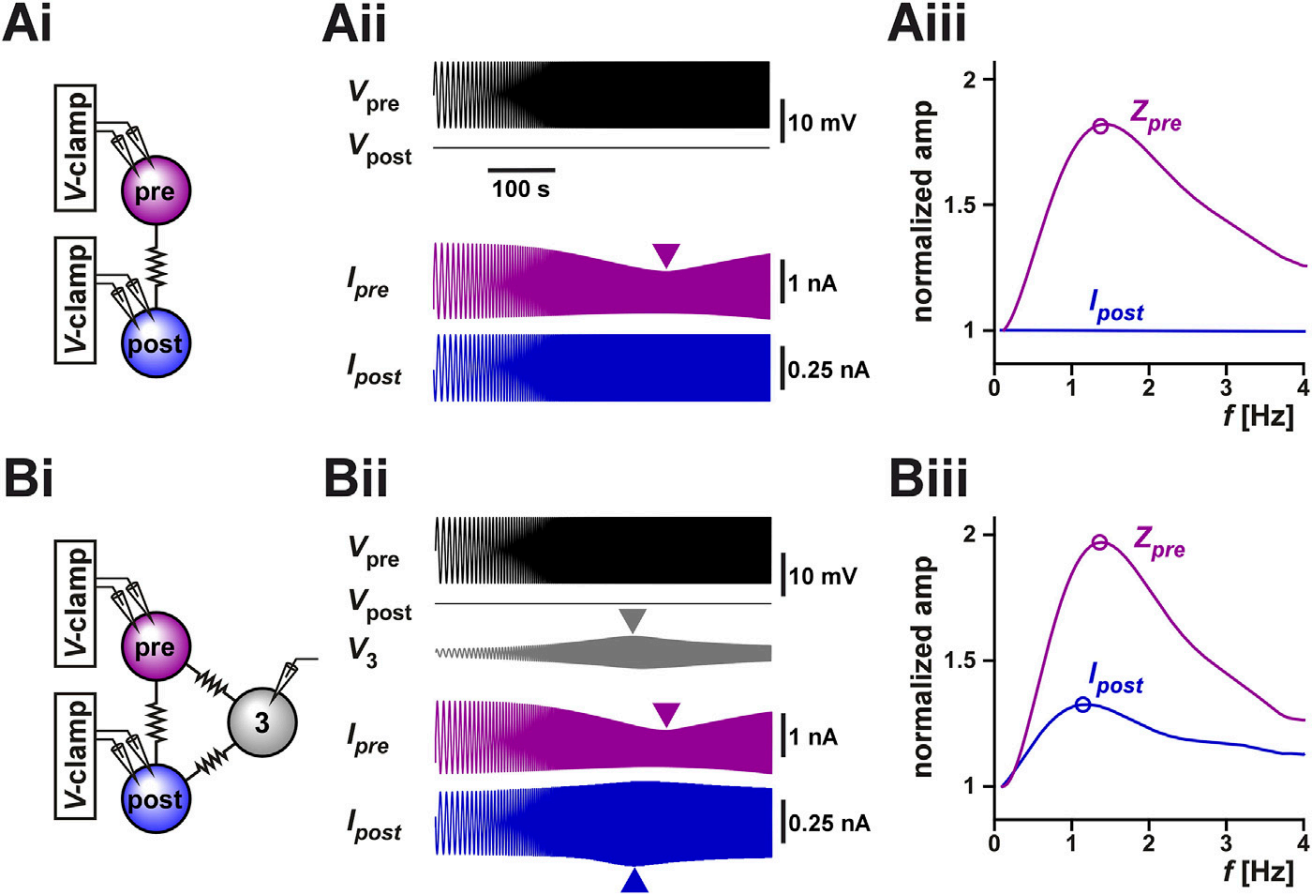
Coupling to a third resonant neuron can produce resonance in the coupling current between two voltage-clamped neurons. **(A)** The coupling current between two identical model neurons with resonant properties was measured in voltage clamp [schematic in **(Ai)**]. The prejunctional neuron was voltage clamped with a ZAP waveform spanning from 0.1 Hz to 4 Hz and voltage range of −60 to −45 mV. The postjunctional neuron was voltage clamped at a holding potential of −60 mV. The postjunctional current amplitude showed no frequency dependence **(Aii)**. As a function of input frequency, the prejunctional impedance shows resonance, but the post junctional current remains constant. For comparison, *Z*_*pre*_ and *I*_*post*_ are normalized to their value at 0.1 Hz. **(B)** The same protocol as A, but the two neurons are both coupled to a third (identical) neuron which is not voltage clamped [schematic in **(Bi)**]. The addition of the third cell leads to a frequency-dependent response in the voltage of the third neuron **(Bii)** and in resonance in the postjunctional current **(Biii)**. For comparison, *Z*_*pre*_ and *I*_*post*_ are normalized to their value at 0.1 Hz.

When both neurons are voltage-clamped, the prejunctional neuron with a fixed-amplitude sinusoidal waveform and the postjunctional neuron at a constant holding voltage (Figure 5Ai), the amplitude of the ionic current change recorded in the postjunctional neuron (*I*_*post*_, which is also the coupling current *I*_*c*_) is proportional to the coupling conductance *G*_*c*_ and independent of any resonant properties of either neuron. This follows from the fact that

$$I_{post}=I_{c}=G_{c}\left(V_{pre}-V_{post}\right)$$

where *V*_*pre*_ and *V*_*post*_ are controlled by voltage clamp and *G*_*c*_ is constant. Although this is an obvious result, it is informative. We demonstrated this in the simulation shown in Figure 5Aii, where the prejunctional model neuron was voltage-clamped with a ZAP function (range −60 to −45 mV) and the prejunctional neuron was held at a steady voltage of −60 mV (parameters for the model given in Table 2). The current (*I*_*pre*_) in the prejunctional neuron showed a minimum, while the current flowing to the postjunctional neuron (*I*_*post*_) did not change with the frequency of the ZAP function (Figure 5Aiii). This is also clear from our calculations for the coupled linear resonators in voltage clamp as shown in Supplementary Appendix SA1 (Supplementary Equation SA1.11).

However, when these two neurons are part of a circuit of electrically coupled neurons, even when both neurons are voltage-clamped, the measurement of *I*_*post*_ may not have a constant amplitude at all input frequencies due to circuit connectivity. For example, if both neurons are electrically coupled to a third neuron whose voltage can vary freely, indirect current flow through the third neuron may affect the amplitude of *I*_*post*_. Indeed, in the pyloric circuit, the two biological PD neurons are electrically coupled to the anterior burster (AB) neuron (Marder and Eisen, 1984) and, in our experiments described above, we did not control or monitor the activity of the AB neuron. It is therefore possible that the apparent resonance we observed in our experimental measurement of *I*_*post*_ (Figure 3Ai) was due to the uncontrolled changes in the voltage of the AB neuron. To test this possibility, we coupled the model neurons of Figure 5Ai to a third neuron with the same resonance properties and ran the same voltage clamp protocol (parameters given in Table 2). Indeed, we observed that even though the pre- and postjunctional neurons were voltage-clamped, the voltage of the third coupled neuron (marked 3 in Figures 5Bi,5Bii) showed a peak at an intermediate frequency. Thus, the resonance of neuron 3 resulted in an apparent resonance in our measured *I*_*post*_, because in this case

$$I_{post}=G_{c}\left(V_{post}-V_{pre}\right)+G_{c}\left(V_{post}-V_{3}\right).$$

A normalized comparison between the impedance profile *V*_*pre*_ and *I*_*post*_ (Figure 5Biii) shows that even when the three neurons are identical in their properties (and therefore have the same isolated resonance frequency), *I*_*post*_ may show resonance at a different frequency, as we had observed in our experimental measurements of Figure 3. Therefore, a potential mechanism for electrical coupling current resonance is through frequency preference inherited from other electrically coupled cells.

### 3.5 Space clamp issues

**FIGURE 6:**
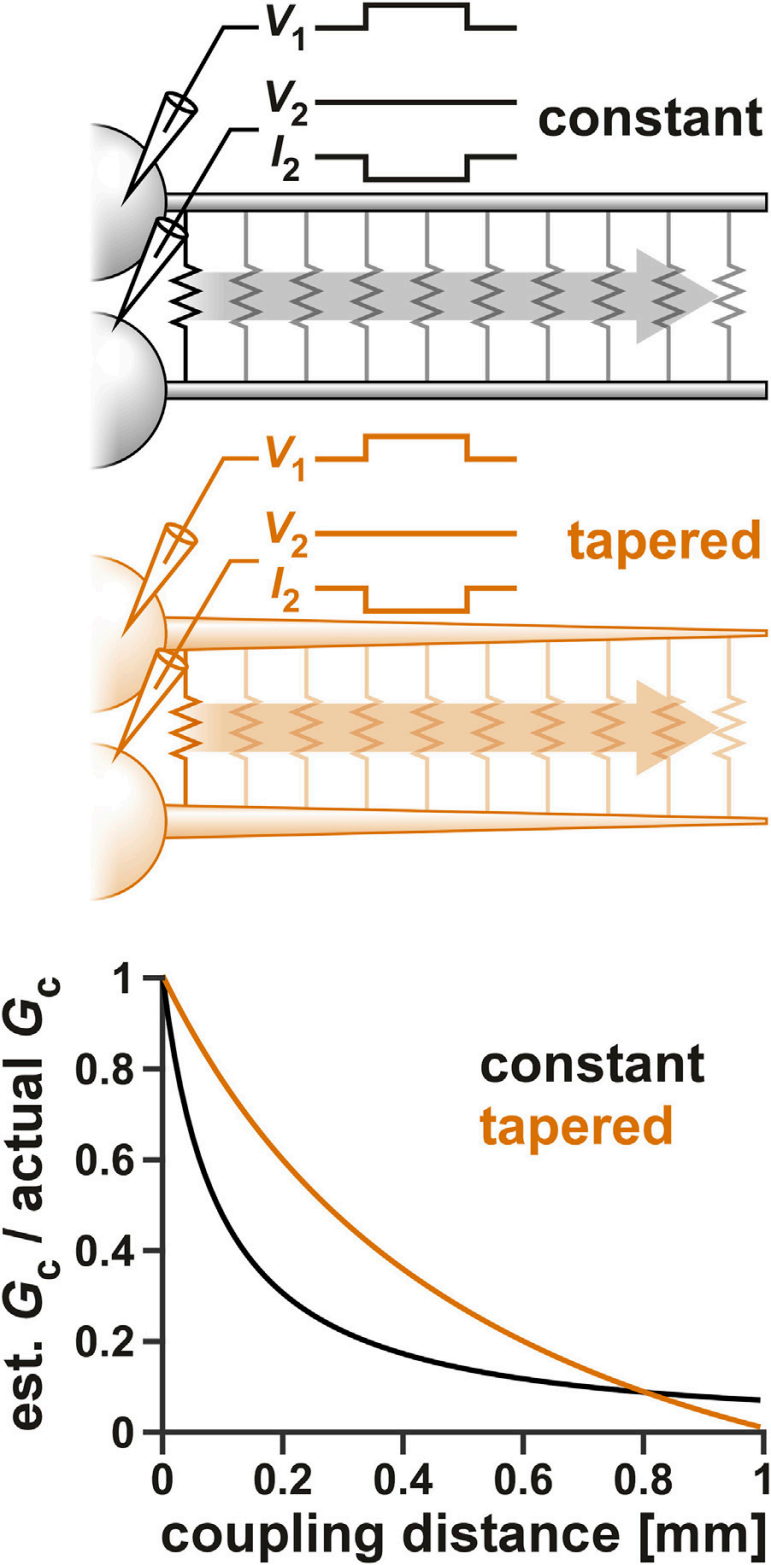
The effect of space clamp error on the measurement of *G*_*c*_. Two ball-and-stick models were examined, one with a standard cylindrical neurite of constant diameter (10 µm), the other with a tapering diameter (20–0.5 µm). Neurite lengths were set to 1,000 µm and the position of the electrical coupling was shifted from the beginning to the end of the neurite, as shown schematically in the top panels. Both somas were voltage clamped and step voltage was applied to one cell. *G*_*c*_ was calculated from the current measured in the second soma. The bottom panel shows the effect of the electrical coupling position on the measurement *G*_*c*_.

Voltage clamp measurements of distal currents are inevitably subject to space clamp errors (To et al., 2022). Considering that gap junctions are most probably located at a distal location to the soma (Otopalik et al., 2019a), it is likely that our voltage-clamp estimate of *G*_*c*_, or even our measurement of Gc resonance is affected by space clamp errors. To examine the extent of such an error, we used a multi-compartmental ball-and-stick model of the PD neurons to estimate the effect of coupling distance from the somatic recording site on *G*_*c*_. Two ball-and-stick models were examined (Figure 6 schematics), one with a standard cylindrical neurite of constant diameter (10 µm), the other with a tapering diameter (20–0.5 µm). As described in a recent detailed study (Otopalik et al., 2019b), the tapered model is a better estimate of the structure of STG neurons.

To estimate the effect of coupling position on *G*_*c*_, the somas of both ball-and-stick model neurons were voltage clamped and a voltage step (from *V*_*hold*_ to *V*_1_) was applied to one (cell 1), while the other (cell 2) was held at a constant voltage (*V*_*hold*_). *G*_*c*_ was then measured as

$$G_{c}=I_{2}/\left(V_{1}-V_{hold}\right).$$

When the position of the coupling was changed along the neurite, the estimated *G*_*c*_ attenuated (Figure 6). The extent of attenuation was much less for the tapered neurite compared to the standard one, except at the very tip, where the tapered neurite became very small in diameter. This was consistent with the findings of Otopalik et al. (2019b) in which they estimated attenuation of a chemical synaptic input. However, considering that the two PD neurons have a relatively strong apparent coupling coefficient, we estimate the coupling position to be no further than halfway along the tapered neurite. Additionally, when we added the ionic currents that produce resonance (Table 2) to these neurons, there was little resonance effect measured in *G*_*c*_ (data not shown).

### 3.6 Potential function of electrical coupling resonance

**FIGURE 7:**
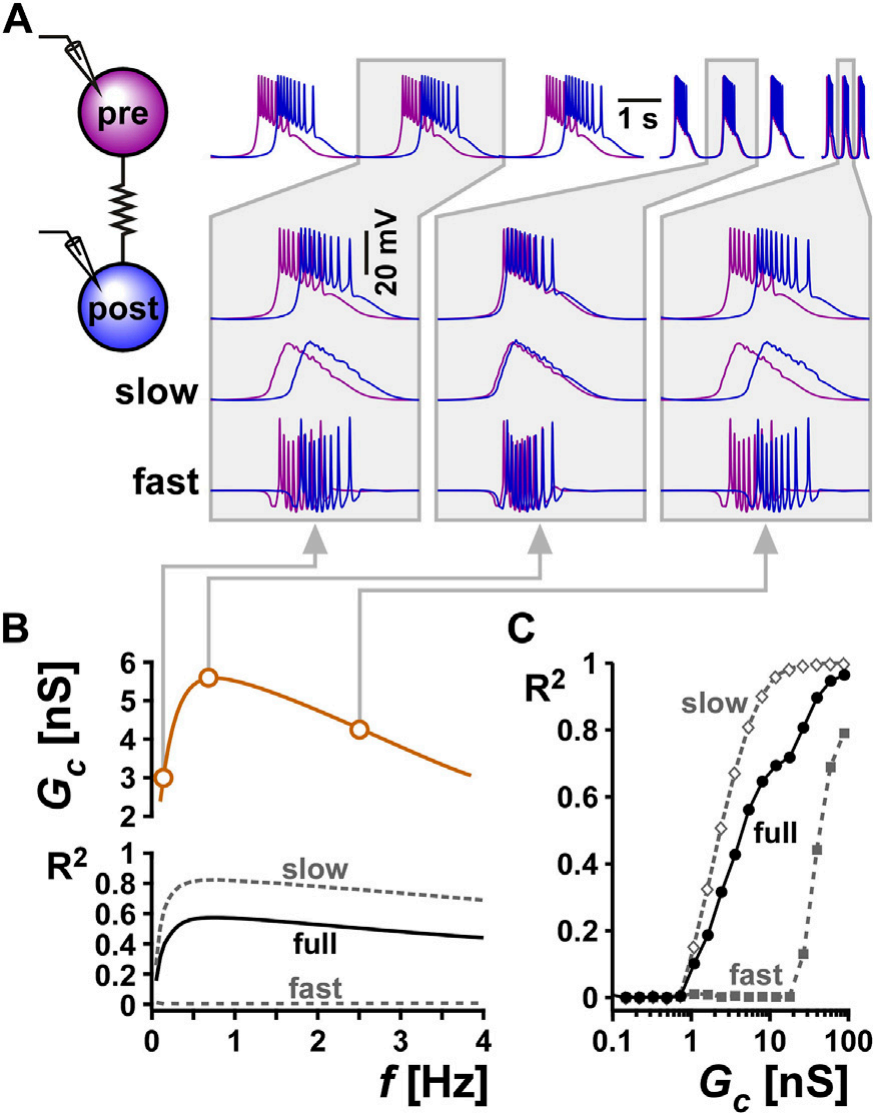
Resonance in the coupling conductance influences the level of synchrony between two model bursting neurons. **(A)** The level of synchrony between two model bursting neurons, coupled with a resonant *G*_*c*_ (schematic), depends on the network oscillation frequency. The three columns show superimposed phase-locked oscillations of two model bursting neurons at three frequencies. The second row is a zoom in to a single burst. The third row shows lowpass filtered traces (slow), highlighting the level of asynchrony of the burst slow waves. The bottom row shows the high pass filtered traces (fast = full - slow), highlighting the lack of synchrony of spiking activity. Gray boxes correspond to frequencies and *G*_*c*_ values as shown in panel **(B) (B)** Coupling conductance is modeled to show resonance at *f* = 0.75 Hz. The level of synchrony between the two coupled neurons, measured as a coefficient of determination *R* ^2^ of their voltage waveforms depends on the network frequency. Changing the network frequency increases synchrony of the slow and full waveforms, but not the fast spiking activity. **(C)** *R* ^2^ increases with the coupling conductance.

We used computational modeling to understand the potential function of resonance in the electrical coupling conductance in this system. We used a computational model of two electrically coupled bursting neurons and chose the parameters of the two neurons to produce bursting oscillations with different cycle frequencies when uncoupled. We then coupled the two neurons and examined the synchronization of their activity at different electrical conductance strengths (Figure 7A). The level of synchronization was measured as the coefficient of determination (*R* ^2^) between the two voltage waveforms (Lane et al., 2016). We measured the synchrony of the full bursting waveforms between the two neurons (full). In addition, we lowpass-filtered the traces to measure the synchrony of only the slow waves (slow), and high pass-filtered to measure the synchrony of only the spiking activity (fast).

To examine the effect of resonance in *G*_*c*_ on the synchrony between the two neurons, we produced a *G*_*c*_ frequency profile similar to that observed experimentally (compare *G*_*c*_ vs. *f* in Figure 7B with Figure 3Bi). Although the two model neurons had different intrinsic burst frequencies, they always oscillated with the same frequency (i.e., they were phase locked) when coupled. To understand the role of *G*_*c*_ resonance, we changed this burst frequency by modifying the intrinsic properties of the bursting neurons (see Methods). We found that when the two cells oscillated at either low or high frequencies, where the *G*_*c*_ was smaller, the slow wave synchrony between the two neurons was smaller (Figure 7C, Bi, Biii). In contrast, when the network frequency matched the *G*_*c*_ resonance frequency, the level of synchronization was maximal (Figure 7C). In contrast to the slow wave, the fast spiking activity of the two neurons was not noticeably altered by frequency. When *G*_*c*_ was kept constant as a function of frequency, then network frequency did not affect the level of synchrony between the two neurons, either in the slow wave or in the spiking activity. The level of synchrony in this case was determined simply by the value of the electrical coupling conductance *G*_*c*_.

## 4 Discussion

Gap junction-mediated electrical coupling between neurons is well known to lead to synchronization of their electrical activity (Gutierrez et al., 2013; Marder et al., 2017; Alcamí and Pereda, 2019; Vaughn and Haas, 2022). However, as a number of modeling studies have shown, in certain conditions it can also promote anti-synchrony (Sherman and Rinzel, 1992; Chow and Kopell, 2000; Bem and Rinzel, 2004). It is commonly assumed that electrical coupling acts primarily as a lowpass filter so that slow voltage changes, such as burst envelopes and subthreshold oscillations, are transmitted more effectively than fast ones such as action potentials (Galaretta and Hestrin, 1998; Connors and Long, 2004; Placantonakis et al., 2006). However, more recent studies that have explored electrical coupling in oscillatory networks have found that the interaction the intrinsic properties of neurons and the electrical coupling could result in a band-pass filtering of the coupling coefficient, such that the coupling coefficient is highest around a “resonance” frequency (Armstrong-Gold and Rieke, 2003; Curti et al., 2012; Stagkourakis et al., 2018). Such bandpass-filtering has been attributed to the properties of voltage-gated ion channels or subthreshold resonance in the coupled neurons (Curti et al., 2012; Alcamí and Pereda, 2019), thus suggesting that the subthreshold resonance frequency can play a significant role in setting the frequency of a network of electrically coupled neurons.

Here, we found similar results in the PD neurons of the crab pyloric circuit. The two PD neurons produce ongoing synchronous bursting activity, are strongly electrically coupled (Figure 1) and show membrane potential resonance (Figure 2; Tohidi and Nadim, 2009). We found that the coupling coefficient of these neurons also shows resonance, but at a much lower frequency than that of their membrane potential resonance (Figure 2). The *CC* resonance frequency, however, was strongly correlated with both that of the pre- and postjunctional neuron. A combined modeling and mathematical analysis showed that although with increased coupling strength the resonance frequencies measured in the coupled neurons converges to the same value, the *CC* resonance frequency does not necessarily fall between these two values (Figure 4C). In fact, our mathematical calculations, based on coupled linear resonators, showed that in response to oscillatory input, *CC* behaves very much like it does in response to a direct current input: It depends on a non-linear combination of the coupling conductance and the impedance of the postjunctional, but not prejunctional, neuron [Eq. 1.1; also see (Alcamí and Pereda, 2019)]. Thus, at least to the first order (linear) approximation, the resonance properties of the prejunctional neuron have no influence on the *CC* resonance frequency, which can fall well outside the range of resonance frequencies of the neurons. This finding is important in the light of the above-mentioned fact that *CC* resonance frequency is often considered to be a determinant of the network oscillation frequency (Curti et al., 2012; Stagkourakis et al., 2018).

The second, perhaps more surprising, finding of our study is that when we measured the current flow between the coupled PD neurons in voltage clamp, we found that the measured coupling was both frequency-dependent in its amplitude and had a resonance frequency distinct from the intrinsic resonance of the PD neurons. For direct current flow between voltage-clamped coupled neurons, this finding inevitably leads to the conclusion that the coupling conductance *G*_*c*_ is frequency-dependent. There are some caveats, however, that should be considered when drawing such a conclusion. First, voltage clamp is often subject to lack of space clamp. If gap junctions that lead to electrical coupling are present in a distal location from the voltage-clamped somata, it is possible that space clamp issues may somehow result in the appearance of frequency-dependence in the coupling current. A structured multi-compartmental ball-and-stick model of the coupled neurons showed that changing the position of the electrical coupling away from the soma reduced the apparent amplitude of the measured *G*_*c*_, but this reduction was only drastic when the coupling position was far from the soma (Figure 6). Additionally, consistent with previous findings showing that the stomatogastric neurons are electrotonically compact (Otopalik et al., 2019b), a tapered neurite showed a much smaller attenuation of the measured *G*_*c*_. Attenuation was only greater when the coupling position was very distal to the soma, an unlikely possibility considering that the biological PD neurons have a large coupling coefficient. However, with the same model we did not find significant resonance in the measured coupling current, indicating that our measured resonance of *G*_*c*_ is unlikely to be due to a space clamp error. The second caveat in drawing a conclusion that *G*_*c*_ is frequency-dependent is that both PD neurons are strongly coupled to the pyloric pacemaker AB neuron, which was neither voltage-clamped nor photo ablated (Miller and Selverston, 1979) here. In fact, a computational model of the three-neuron coupled circuit showed that a free-running AB neuron may indeed result in an apparent resonance of the coupling current measured between the two PD neurons (Figure 5Biii). Although we did not resolve the caveat of coupling to additional neurons in the current study, our unpublished results indicate that there is a possibility that frequency-dependence may in fact in part be inherent to the electrical coupling conductance. These findings showed that peptide neuromodulators that activate the same ionic current in the pyloric pacemaker neurons have an opposite effect on shifting both the frequency and amplitude of resonance in the coupling current (Li et al., 2017). This result cannot be explained by coupling to a free-running AB neuron which is modulated the same way by the two peptides. Consequently, the gap junction channels may in fact have kinetics that allows for bandpass filtering. Although it is know that current flow through gap junctions may have complex and functional voltage-dependent properties (examples in Coleman et al., 1995; Vaughn and Haas, 2022), to our knowledge, such a frequency-dependent filtering property of gap junctions has not been previously reported.

Previous studies have suggested that different resonant properties of different circuit components collectively influence network frequency (Lovett-Barron et al., 2017). However, it remains to be determined to what extent *CC* or *G*_*c*_ resonance interacts with other frequency-dependent properties of a network. We showed, however, that resonance in *G*_*c*_ or the coupling current would amplify the resonance properties of *CC* (Figure 4D). In addition, one functional consequence of the frequency-dependence of the coupling is intuitively clear if the network frequency may be subject to context-dependent changes. We demonstrated this using a coupled network of two intrinsically distinct model neurons. Although at all frequencies tested, the two neurons remained phase locked, their degree of synchronization was effectively determined by the frequency-dependent properties of the coupling conductance (Figure 7). In an oscillatory network such as the crab pyloric network, where network frequency depends on multiple factors including neuromodulation and temperature, it is reasonable to assume that the degree of synchronization between the PD neurons may be influenced indirectly by the factors that modify network frequency. Although the experimental verification of these functional consequences remains to be performed, our combined experimental and modeling findings indicate that the resonance properties of electrical coupling may play a central role in shaping the output of oscillatory networks.

## Data Availability

The raw data supporting the conclusion of this article will be made available by the authors, without undue reservation.
