# Supplementary material for: Distinct mechanisms underlie electrical coupling resonance and its interaction with membrane potential resonance
Source: Front Syst Biol. 2023 Mar 8;3:1122433. doi: 10.3389/fsysb.2023.1122433 (PMC12341970; doi:10.3389/fsysb.2023.1122433)
Supplement: Supplementary file 1 [file DataSheet1.docx]

# Appendix 1

## Electrically coupled linear cells receiving oscillatory inputs

The general form of the electrically coupled two-cells network model we use is given by

$$\begin{matrix} C\frac{dV_{1}}{dt}=-g_{L,1}V_{1}-g_{R,1}w_{1}+G_{c}(V_{2}-V_{1})+I_{1}(t) \\ \tau_{1}\frac{dw_{1}}{dt}=V_{1}-w_{1} \\ C\frac{dV_{2}}{dt}=-g_{L,2}V_{2}-g_{R,2}w_{2}+G_{c}(V_{1}-V_{2})+I_{2}(t) \\ \tau_{2}\frac{dw_{2}}{dt}=V_{2}-w_{2} \end{matrix}$$

The dynamics of the individual cells in system are the linearization of biophysically plausible (conductance-based) models around the resting potentials (Richardson et al., 2003; Rotstein and Nadim, 2014; 2019). For *k* = 1,2, *V_k_* represents the membrane potential for the two cells and measures deflections from a resting potential (which here would be equal to 0), *w_k_* represents the corresponding recovery variables after linearization, *t* (ms) is time, *C* is the specific capacitance, *g_L,k_* are the linearized leak conductances, *g_R,k_* are the linearized ionic conductances, *G_c_* (µS/cm^2^) is the electrical coupling conductance, and *I_k_* are time-dependent currents. In this Appendix, we are using dimensional parameters, with time in ms, frequencies in Hz, voltages and recovery variables in mV, capacitance in µF/cm^2^, conductances in µS/cm^2^ and currents in mA/cm^2^.

In current-clamp (I-clamp),

$$I_{k}(t)=I_{app,k}+A_{in,k}\sin(2\pi ft/1000)$$

for *k* = 1, 2, where *A_in,k_* and *f* are the externally-applied amplitudes and frequencies and *I_app,k_* is a constant (DC) current. In voltage-clamp (V-clamp),

$$V_{k}(t)=V_{app,k}+A_{in,k}\sin(2\pi ft/1000)$$

for *k* = 1, 2, where *A_in,k_* and *f* are as above and *V_app,k_* is a constant holding voltage. In the cases we consider here, except for the uncoupled cells (*G_c_* = 0) that we use as a reference case to establish the resonant properties of the individual cells, only one cell (cell 1) receives an oscillatory input (regardless of whether it is in I- or V-clamp). Therefore, we refer to cells 1 and 2 as the pre- and postjunctional cells, respectively. To simplify the notation, we define

$$\begin{matrix} a_{k}=-\frac{g_{L,k}}{C},\text{ }b_{k}=-\frac{g_{R,k}}{C},\text{ }c_{k}=\frac{1}{\tau_{k}},\text{ }d_{k}=-\frac{1}{\tau_{k}}, \\ \gamma_{c}=\frac{G_{c}}{C},\text{ }\hat{I}_{k}=\frac{I_{k}}{C},\text{ }\omega=\frac{2\pi f}{1000}. \end{matrix}$$

Substitution into system yields

$$\begin{matrix} \frac{dV_{1}}{dt}=a_{1}V_{1}+b_{1}w_{1}+\gamma_{c}(V_{2}-V_{1})+\hat{I}_{1}(t) \\ \frac{dw_{1}}{dt}=c_{1}V_{1}+d_{1}w_{1} \\ \frac{dV_{2}}{dt}=a_{2}V_{2}+b_{2}w_{2}+\gamma_{c}(V_{2}-V_{1})+\hat{I}_{2}(t) \\ \frac{dw_{2}}{dt}=c_{2}V_{2}+d_{2}w_{2}. \end{matrix}$$

For use below, we further define the determinants and traces of the matrices (for *k* = 1, 2) of the coefficients of the linear system:

$$\begin{matrix} \Delta_{k}=a_{k} d_{k}-b_{k} c_{k}=\frac{g_{L,k}+g_{R,k}}{C \tau_{k}} \\ \Gamma_{k}=a_{k}+d_{k}=-\frac{g_{L,k} \tau_{k}+C}{C \tau_{k}}. \end{matrix}$$

## Response of the uncoupled cells to oscillatory inputs: cellular impedances and inverse admittances

Here we consider *γ_c_* = 0 and $\hat{I}_{k}(t)$ given by , with *A_in_*_,1_ = *A_in_*_,2_ = *A_in_* and *I_app_*_,1_ = *I_app_*_,2_ = 0. The impedances of the individual uncoupled cells, as described previously (Richardson et al., 2003; Rotstein and Nadim, 2014), are given by

$$\mathbf{Z}_{\mathbf{k}}(\omega)=\frac{(-d_{k}+i \omega)}{a_{k} d_{k}-b_{k} c_{k}-\omega^{2}-i (a_{k}+d_{k}) \omega}=\frac{(-d_{k}+i \omega)}{\Delta_{k}-\omega^{2}-i \omega\Gamma_{k}}.$$

The impedance amplitudes and phases (phase-shifts) are given, respectively, by

$$Z_{k}^{2}(\omega)=\frac{d_{k}^{2}+\omega^{2}}{(\Delta_{k}-\omega^{2})^{2}+\Gamma_{k}^{2} \omega^{2}}\text{ and }\Phi(\omega)=\tan^{-1}\frac{(\Delta_{k}-\omega^{2})-\Gamma_{k} d_{k}}{(\Delta_{k}-\omega^{2}) d_{k}+\Gamma_{k} \omega^{2}} \omega.$$

Therefore, the solutions to equations for the uncoupled neurons, each receiving sinusoidal input currents, are given by

$$\begin{matrix} V_{1}(t,\omega)=Z_{1}(\omega)A_{in}\sin[ \omega t-\Phi_{1}(\omega) ] \\ V_{2}(t,\omega)=Z_{2}(\omega)A_{in}\sin[ \omega t-\Phi_{2}(\omega) ]. \end{matrix}$$

These calculations correspond to I-clamp. In V-clamp, *V_k_*(*t*) is given by *A_in_*_,1_ = *A_in_*_,2_ = *A_in_* and *V_app_*_,1_ = *V_app_*_,2_ = 0. Since the system is linear, as described previously (Rotstein and Nadim, 2019), the admittances are given by

$$\mathbf{Y}_{\mathbf{k}}(\omega)=\frac{1}{\mathbf{Z}_{\mathbf{k}}(\omega)}$$

and

$$\hat{I}_{K}(t,\omega)=\frac{1}{Z_{k}(\omega)}A_{in}\sin[\omega t+\Phi_{k}(\omega)]$$

for *k* = 1, 2. Note that for nonlinear systems, the equality between the impedance (measured in I-clamp) and the inverse admittance (measured in V-clamp) does not generally hold Rotstein, 2019 #4352}.

In order to compute the impedances, we used the complex exponential expression for $\hat{I}_{k}(t)=A_{in}\exp(i\omega t)$ and assumed (from linearity) that the stationary solutions to system are given by

$$\begin{matrix} V_{1,out}=\mathbf{A}_{\mathbf{1},\mathbf{out}}(\omega)\exp(i\omega t) \\ V_{2,out}=\mathbf{A}_{\mathbf{2},\mathbf{out}}(\omega)\exp(i\omega t) \\ w_{1,out}=\mathbf{B}_{\mathbf{1},\mathbf{out}}(\omega)\exp(i\omega t) \\ w_{2,out}=\mathbf{B}_{\mathbf{2},\mathbf{out}}(\omega)\exp(i\omega t). \end{matrix}$$

We then substituted these expressions into equations and computed the coefficients

$$\begin{matrix} \mathbf{A}_{\mathbf{1},\mathbf{out}}(\omega)=\mathbf{Z}_{\mathbf{1}}(\omega)A_{in} \\ \mathbf{A}_{\mathbf{2},\mathbf{out}}(\omega)=\mathbf{Z}_{\mathbf{2}}(\omega)A_{in}. \end{matrix}$$

## Response of the electrically coupled cells to oscillatory inputs solely to the prejunctional cell (cell 1) in I-clamp

Here we assume that $\hat{I}_{1}(t)$is a sinusoidal input current of the form with *A_in_*_,1_ = *A_in_*, *I_app_*_,1_ = 0 and $\hat{I}_{2}(t)=0$. Equivalently, $\hat{I}_{1}(t)=A_{in}\exp(i\omega t)$ and $\hat{I}_{2}(t)=0$. Substitution of the formal solutions into equations yields

$$\begin{matrix} \left[\frac{1}{\mathbf{Z}_{\mathbf{1}}(\omega)}+\gamma_{c} \right]\mathbf{A}_{\mathbf{1}}(\omega)-\gamma_{c}\mathbf{A}_{\mathbf{2}}(\omega)=A_{in}, \\ -\gamma_{c} \mathbf{A}_{\mathbf{1}}(\omega)+\left[\frac{1}{\mathbf{Z}_{\mathbf{2}}(\omega)}+\gamma_{c}\right]\mathbf{A}_{\mathbf{2}}(\omega)=0. \end{matrix}$$

By solving this algebraic system, we obtain

$$\begin{matrix} \mathbf{A}_{\mathbf{1}}(\omega)=\frac{\mathbf{Z}_{\mathbf{2}}^{-1}(\omega)+\gamma_{c}}{[\mathbf{Z}_{\mathbf{1}}^{-1}(\omega)+\gamma_{c}] [\mathbf{Z}_{\mathbf{2}}^{-1}(\omega)+\gamma_{c}]-\gamma_{c}^{2}} A_{in} \\ \mathbf{A}_{\mathbf{2}}(\omega)=\frac{\gamma_{c}}{[\mathbf{Z}_{\mathbf{1}}^{-1}(\omega)+\gamma_{c}] [\mathbf{Z}_{\mathbf{2}}^{-1}(\omega)+\gamma_{c}]-\gamma_{c}^{2}} A_{in}. \end{matrix}$$

Therefore, the impedances of the coupled cells are given by

$$\begin{matrix} \mathbf{Z}_{\mathbf{1},\mathbf{c}}(\omega)=\frac{\mathbf{Z}_{\mathbf{2}}^{-1}(\omega)+\gamma_{c}}{[\mathbf{Z}_{\mathbf{1}}^{-1}(\omega)+\gamma_{c}] [\mathbf{Z}_{\mathbf{2}}^{-1}(\omega)+\gamma_{c}]-\gamma_{c}^{2}} \\ \mathbf{Z}_{\mathbf{2},\mathbf{c}}(\omega)=\frac{\gamma_{c}}{[\mathbf{Z}_{\mathbf{1}}^{-1}(\omega)+\gamma_{c}] [\mathbf{Z}_{\mathbf{2}}^{-1}(\omega)+\gamma_{c}]-\gamma_{c}^{2}}. \end{matrix}$$

The corresponding solutions to system are given by

$$\begin{matrix} V_{1,c}(t,\omega)=Z_{1,c}(\omega)A_{in}\sin[ \omega t-\Phi_{1,c}(\omega) ] \\ V_{2,c}(t,\omega)=Z_{2,c}(\omega)A_{in}\sin[ \omega t-\Phi_{2,c}(\omega) ], \end{matrix}$$

where *Z_k,c_*(*ω*) and Φ*_k,c_*(*ω*) are the amplitudes and phases of **Z***_k,c_*(*ω*) for *k* =1, 2. We refer to *Z*_1_*_,c_* as the prejunctional impedance and to *Z*_2_*_,c_* as the postjunctional impedance (*Z_pre_* and *Z_post_* respectively in Table 1).

These calculations assume the postjunctional cell (cell 2) is I-clamped. If, instead, the postjunctional cell is V-clamped, (*V*_2_(*t*) = *V_app_*_,2_), then

$V_{1}(t,\omega)=-\gamma_{c}V_{app,2}\left(\frac{\Delta_{1}}{d_{1}}-\gamma_{c}\right)^{-1}+\mathbf{Z}_{\mathbf{1},\mathbf{c}}(\omega)\exp(i\omega t)$,

$$I_{2}(t,\omega)=\left(-\frac{\Delta_{2}}{d_{2}}+\gamma_{c}\right)V_{app,2}-\gamma_{c}A_{in}\exp(i\omega t)$$

with

$$\mathbf{Z}_{\mathbf{1},\mathbf{c}}(\omega)=\frac{(-d_{1}+i \omega)}{\Delta_{1}-\gamma_{c}d_{1}-\omega^{2}-i \omega(\Gamma_{1}-\gamma_{c})}.$$

Therefore

$$V_{1}(t,\omega)=-\gamma_{c}V_{app,2}\left(\frac{\Delta_{1}}{d_{1}}-\gamma_{c}\right)^{-1}+Z_{1,c}(\omega)A_{in}\sin[ \omega t-\Phi_{1,c}(\omega) ]$$

with

$$Z_{1,c}(\omega)=\sqrt{\frac{d_{1}^{2}+\omega^{2}}{(\Delta_{1}-\omega^{2}-\gamma_{c}d_{1})^{2}+(\Gamma_{1}-\gamma_{c})^{2} \omega^{2}}}$$

and

$$\Phi_{1,c}(\omega)=\tan^{-1}\frac{(\Delta_{1}-\omega^{2}-\gamma_{c} d_{1})-(\Gamma_{1}-\gamma_{c}) d_{1}}{(\Delta_{1}-\omega^{2}-\gamma_{c} d_{1}) d_{1}+(\Gamma_{1}-\gamma_{c}) \omega^{2}} \omega.$$

From ,

$$\mathbf{Y}_{\mathbf{2},\mathbf{c}}(\omega)=\frac{1}{\gamma_{c}}.$$

## The coupling coefficient, *CC*

The coupling coefficient (*CC*; Table 1) is given by

$$\begin{matrix} CC=\frac{Z_{2,c}(\omega)}{Z_{1,c}(\omega)}=\left| \frac{\mathbf{Z}_{\mathbf{2},\mathbf{c}}(\omega)}{\mathbf{Z}_{\mathbf{1},\mathbf{c}}(\omega)} \right|=\frac{\gamma_{c}}{| \mathbf{Z}_{\mathbf{2}}^{-1}(\omega)+\gamma_{c} |}=\frac{\gamma_{c} Z_{2}(\omega)}{| 1+\gamma_{c} \mathbf{Z}_{\mathbf{2}}(\mathbf{\omega}) |} \\ =\gamma_{c} \sqrt{\frac{d_{2}^{2}+\omega^{2}}{(\Delta_{2}-\omega^{2}-\gamma_{c}d_{2})^{2}+(\Gamma_{2}-\gamma_{c})^{2} \omega^{2}}}. \end{matrix}$$

Formally, *CC* can be expressed in terms of the impedance of the isolated postjunctional cell and is independent of the impedance of the prejunctional cell.

If *Z*_2_(*ω*) acts as a low-pass filter (i.e., *b*_2_ = 0), then

$$CC=\frac{\gamma_{c}}{\sqrt{(a_{2}-\gamma_{c})^{2}+\omega^{2}}}$$

is also a low-pass filter.

## Response of the electrically coupled cells to oscillatory inputs solely to the prejunctional cell (cell 1) in V-clamp

Here we assume that *V*_1_(*t*) is a sinusoidal input of the form with *A_in_*_,1_ = *A_in_* and *V_app_*_,1_ = 0 and *V*_2_ = *V_app_*_,2_ at a constant value. Equivalently, $V_{1}(t)=A_{in}\exp(i\omega t)$. Substitution of these expressions into yields

$$\begin{matrix} I_{1}=-\gamma_{c}V_{app,2}+\left(\frac{1}{\mathbf{Z}_{\mathbf{1}}(\omega)}+\gamma_{c}\right)A_{in}\exp(i\omega t) \\ I_{2}=-\left(\frac{\Delta_{2}}{d_{2}}-\gamma_{c}\right)V_{app,2}-\gamma_{c} A_{in}\exp(i\omega t). \end{matrix}$$

Therefore, the admittance of the coupled neurons are given by

$$\begin{matrix} \mathbf{Y}_{\mathbf{1},\mathbf{c}}^{-\mathbf{1}}(\omega)=\left(\frac{1}{\mathbf{Z}_{\mathbf{1}}(\omega)}+\gamma_{c}\right)^{-1}=\frac{\mathbf{Z}_{\mathbf{1}}(\omega)}{1+\gamma_{c} \mathbf{Z}_{\mathbf{1}}(\omega)}, \\ Y_{1,c}^{-1}(\omega)=\left|\mathbf{Y}_{\mathbf{1},\mathbf{c}}^{-\mathbf{1}}(\omega)\right|=\frac{Z_{1}(\omega)}{|1+\gamma_{c} \mathbf{Z}_{\mathbf{1}}(\omega)|}=\sqrt{\frac{d_{1}^{2}+\omega^{2}}{(\Delta_{1}-\omega^{2}-\gamma_{c}d_{1})^{2}+(\Gamma_{1}-\gamma_{c})^{2} \omega^{2}}}, \end{matrix}$$

and

$$\mathbf{Y}_{\mathbf{2},c}^{-\mathbf{1}}(\omega)=\frac{1}{\gamma_{c}}\text{ or }\mathbf{Y}_{\mathbf{2},c}(\omega)=\gamma_{c}.$$

# References

Richardson, M.J., Brunel, N., and Hakim, V. (2003). From subthreshold to firing-rate resonance. *J Neurophysiol* 89(5)**,** 2538-2554. doi: 10.1152/jn.00955.2002

00955.2002 [pii].

Rotstein, H.G., and Nadim, F. (2014). Frequency preference in two-dimensional neural models: a linear analysis of the interaction between resonant and amplifying currents. *J Comput Neurosci* 37(1)**,** 9-28. doi: 10.1007/s10827-013-0483-3.

Rotstein, H.G., and Nadim, F. (2019). Frequency-dependent responses of neuronal models to oscillatory inputs in current versus voltage clamp. *Biol Cybern* 113(4)**,** 373-395. doi: 10.1007/s00422-019-00802-z.
